# Analysis of Practical Identifiability of a Viral Infection Model

**DOI:** 10.1371/journal.pone.0167568

**Published:** 2016-12-30

**Authors:** Van Kinh Nguyen, Frank Klawonn, Rafael Mikolajczyk, Esteban A. Hernandez-Vargas

**Affiliations:** 1 Systems Medicine of Infectious Diseases, Department of Systems Immunology and Braunschweig Integrated Centre of Systems Biology, Helmholtz Centre for Infection Research, Braunschweig, Germany; 2 Epidemiology Department, Ho Chi Minh University of Medicine and Pharmacy, Ho Chi Minh, Vietnam; 3 PhD Programme “Epidemiology”, Braunschweig-Hannover, Germany; 4 Biostatistics, Helmholtz Centre for Infection Research, Braunschweig, Germany; 5 Department of Computer Science, Ostfalia University, Wolfenbüttel, Germany; 6 Epidemiological and Statistical Methods, Helmholtz Centre for Infection Research, Braunschweig, Germany; 7 German Centre for Infection Research, site Hannover-Braunschweig, Germany; 8 Hannover Medical School, Hannover, Germany; 9 [Institute of] Medical Epidemiology, Biometry and Informatics, Martin-Luther University Halle-Wittenberg, Germany; Friedrich-Alexander-Universitat Erlangen-Nurnberg, GERMANY

## Abstract

Mathematical modelling approaches have granted a significant contribution to life sciences and beyond to understand experimental results. However, incomplete and inadequate assessments in parameter estimation practices hamper the parameter reliability, and consequently the insights that ultimately could arise from a mathematical model. To keep the diligent works in modelling biological systems from being mistrusted, potential sources of error must be acknowledged. Employing a popular mathematical model in viral infection research, existing means and practices in parameter estimation are exemplified. Numerical results show that poor experimental data is a main source that can lead to erroneous parameter estimates despite the use of innovative parameter estimation algorithms. Arbitrary choices of initial conditions as well as data asynchrony distort the parameter estimates but are often overlooked in modelling studies. This work stresses the existence of several sources of error buried in reports of modelling biological systems, voicing the need for assessing the sources of error, consolidating efforts in solving the immediate difficulties, and possibly reconsidering the use of mathematical modelling to quantify experimental data.

## Introduction

Mathematical modelling approaches have been playing a central role to describe many different real life applications [1]. In biological and medical research, benefits from mathematical modelling are not only generating and validating hypotheses from experimental data but also simulating these models has led to testable experimental predictions [2]. Presently, quantitative modelling is among the renowned methods used to study several aspects of viral infection diseases from virus-host interactions to complex immune responses systems and therapies [3–6], cancer [7], and gene networks [8].

Mechanistic models aim to mimic biological mechanisms that embody some essential and exciting aspects of a particular disease. These can serve either as pedagogical tools to understand and predict disease progression, or act as the objects of further experiments. However, mathematical models at heart are incomplete, and the same process can be modelled differently, if not competitively. Inherently, a model should not be accepted or proven as a correct one, but should only be seen as the least invalid model among the alternatives. This selection process can be done, for example, by fitting the models to experimental data and comparing the models’ goodness-of-fit criterion e.g., [9–11]. Alternatively, modelling can be used as “thought experiments” without considering parameter fitting to experimental data. For instance, on the basis of various simple mathematical models, the hypothesis that the immune activation determines the decline of memory CD4+ T cells in HIV infection can be rejected [12].

Assuming there exists a model that represents properly the problem at hand, model fitting to experimental data is still subject to a number of factors that can distort parameter estimates. For instance, mathematical models are often non-linear in structure and their parameters can be strongly correlated, posing troublesome issues to parameter estimation procedures, e.g., parameter identifiability [13] or parameter sensitivity [14]. These problems aggravate as experimental data are often sparse, scarce, and vary by orders of magnitude [9, 13, 15–19]. Consequently, accurate estimation of biological parameters is inherently problematic, if not impossible [14].

**Fig 1:**
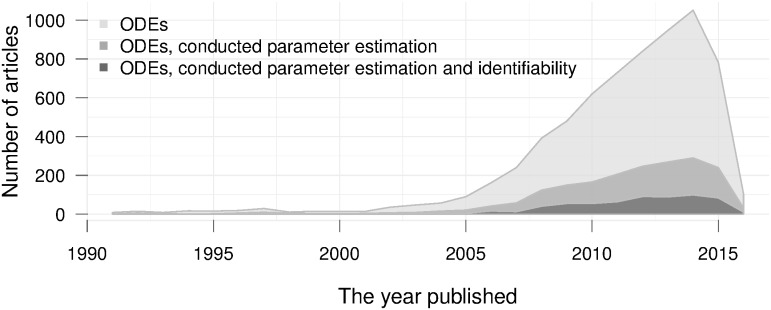
Trends of applied mathematical modelling in biological and medical research over the last decades. **ODEs**: papers that used ordinary different equations. The data were queried directly from PubMed Central (PMC) database (see S5 Text). The curtailment in the recent years is due to the embargo period of publishing to PMC database [25].

Nevertheless, above issues are not *de facto* worrisome barriers if the primary purpose is to evaluate working hypotheses [14, 20]. In this case, finding a working set or a plausible range of parameter values can be deemed sufficient. However, doubts arise when mathematical models are used explicitly to extract biological meaning of model parameters, which is more evident and likely to happen in those applications where the prediction aspect is inferior. For example, modelling studies on acute diseases with predictable dynamics, such as influenza virus infection [3, 4, 21–24], have been pursuing the quantification of parameters rather than prediction. Additionally, the conventional practice of making use of the formerly estimated parameters in mathematical modelling could propagate the potential inaccurate parameters to the subsequent works, weaken the validity of parameter estimates and consequently reduce the model merit. Several approaches have been offered to provide assessments and understanding on the soundness of the estimated parameters, e.g., [13, 14, 19]. However, in fact, the booming works on mathematical models in biological and medical research over the last years have been accompanied with a disproportionately low amount of assessments on parameter validity (Fig 1). This quantitative recapitulation of biological parameters without a doubt has raised concerns in the scientific community, provoking questions on the appropriateness of the approach.

To keep modelling biological systems from being mistrusted, an important step is to recognize potential sources of error and to assess them in publications. This practice will not only increase the parameter credibility but also save the future works from repeating the same mistakes. In this paper, numerical simulations of a popular mathematical model in viral infection research are used to exemplify various sources of error that exist even when a correct and structurally identifiable model is being used to fit designated data. Thus, this paper provides different angles of the limitations in quantifying biological parameters in mathematical modelling. The results are entrusted to foster rich conversations in the scientific community towards efforts in posing good practice guidelines; and hopefully, to bring changes in the school of thoughts of mathematical modelling.

## Materials and Methods

Studying sources of error from real experimental data can be debatable [26] as it limits the abilities to examine a key issue being studied in isolation from confounding factors. Therefore, a typical mathematical model structure was used to generate synthetic experimental data in various scenarios that resemble data in practice. Each scenario was designed to address a particular source of error while other potentially influential factors were kept under control. Parameter estimation procedures were then applied to recover the model parameters, isolating the impact of each factor on the parameter estimates. The errors of the parameter estimates were evaluated in terms of differences between the estimated values and the original simulation parameters.

To this end, the target-cell limited model presented in [27] was adopted owing to its role as the core component of more than a hundred publications in virus research, e.g., influenza [11, 21, 22, 27–30], HIV [31–34], and Ebola [35] among others [5]. The model includes three compartments: uninfected cells (U), infected cells (I), and viral load (V). It consists of four parameters with partially observable dynamics, i.e., only the viral load dynamics is measured. The model reads as follows

dU/dt=−βUV, (1)

dI/dt=βUV−δI, (2)

dV/dt =pI−cV, (3)

where the parameters to be estimated from experimental data are *β*, *δ*, *p*, and *c*, which represent the rates of effective infection, infected cell death, viral replication, and viral clearance, respectively. Structural identifiability analyses of the model have shown that the four model parameters can be identified if initial conditions are known [9]. In this model, virus infection is limited only by the availability of the uninfected cells. The uninfected cells are infected by the viruses and become infectious, consequently, these infectious cells are able to release virus particles.

**Fig 2:**
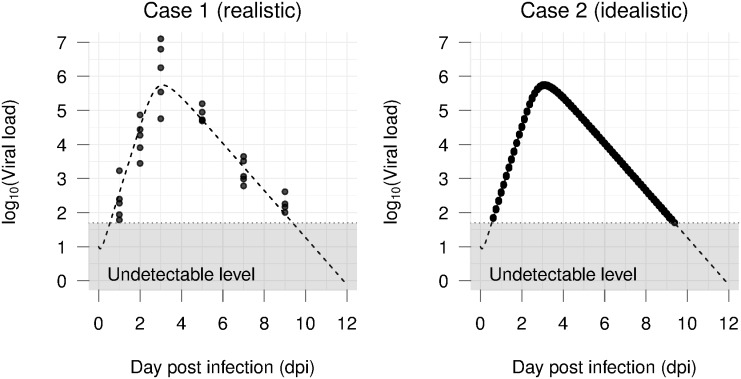
Examples of generated data of iav. **Left**: A realistic data set with five replicates per time point and measures at irregular time points. **Right**: An idealistic data set with thirty replicates per time point and measures every three hours. The measurement errors are assumed following *N*(0, 0.25), *N*(0, 0.01) on log base ten scale for Case 1 and Case 2, respectively.

Influenza A virus (IAV) infection dynamics data were considered as reference because of its acute progression nature. It has also been described and modelled extensively [4]. Simulation scenarios were differentiated in number and spacing of sampling time points, number of replicates per time point, and whether a certain experimental setting is known. The reference parameters were chosen to have synthetic data that closely resemble typical experimental observations. In particular, iav infection kinetics comprises a quick viral replication and a peak around the 2^nd^ and 3^rd^ dpi [3, 4, 21]. The viral load stays in a plateau shortly before it declines to an undetectable level below lod at day 7–12 onwards [3, 4, 21]. The half-life of infected cells (*δ* = 1.6) was assumed shorter than that of healthy epithelial cells [36]. The viral clearance rate (*c* = 3.7) was approximated to that presented in [37]. The remaining two parameters were chosen such that the viral load exponentially increases and peaks at 3^rd^ dpi then declines to the undetectable level at 9^th^ dpi, i.e., *β* = 10^−5^, *p* = 5. Unless stated otherwise, the initial number of uninfected cells, infected cells and virus inoculum are assumed known with *U*_0_ = 10^6^, *I*_0_ = 0, and *V*_0_ = 10, respectively. To reflect the lod, generated data points that are smaller than 50 pfu/ml were removed. The measurement error is assumed to be normally distributed on log base ten, i.e., ∼*N*(0, 0.25) unless otherwise stated. Fig 2 visualizes the two examples of the generated data that mimic a realistic and an idealistic situation. Outlines of the detailed variations of the generated data are shown in Table 1.

**Table 1:** Summary of different simulation settings.

Illustrating error in	Time scheme*	Replicates**	Noise***	Initial condition	Algorihtms
a. Initial conditions	t3	30	N(0,0.01)	Vary	DE [43]
b. Measurement error	t3	30	Vary	Fixed	DE [43]
c. Effect of time scheme	Vary	10	N(0,0.25)	Fixed	L-BFGS-B [44]
d. Effect of number of replicates	t24	Vary	N(0,0.25)	Fixed	L-BFGS-B [44]
e. Algorithms	tn1	5	N(0,0.25)	Fixed	Vary
f. Robust estimation	t24	10	N(0,0.25)	Fixed	Vary
g. Polynomial estimate of *V*_0_	Vary	Vary	N(0,0.25)	Not used	GAM [38]
Parameters	Reference	Lower bound^+^	Upper bound^+^		
*β*	10^−5^	10^−7^	10^−3^		
*δ*	1.6	10^−2^	10^2^		
p	5	10^0^	10^2^		
c	3.7	10^−1^	10^2^		
Initial condition	*U*_0_ = 10^6^	*V*_0_ = 10	*I*_0_ = 0		

*t3 to t24 indicate regular sampling time in every 3 to 24 hours; tn1 indicates sampling time similar to that of [11], i.e., at day 1, 2, 3, 5, 7, 9, tn2 is t24 without measurement at the peak day (3rd) and the endpoint (8th);

**per time point;

***on log 10 scale;

^+^for Differential Evolution (DE) algorithm and L-BFGS-B [44].

To illustrate the error of the recovered parameter estimates, their profile likelihood [13], bias, and variance were computed. In this paper, parameters profiles are used to show if a parameter was identified without error. Each scenario was simulated with a large number of times to compute the bias and the variance [38] of the estimator. Posterior samples [39], weighted bootstrapping samples [40], and the confidence interval based on the approximate observed Fisher information [41] are used to express uncertainty measures where relevant. Simulations were performed using R 3.0.2 [42] and its provided packages when available, otherwise the computations were done with self-written R code.

## Results

### Choices of initial conditions skew parameter estimates

Generally, the model’s initial conditions can be included in parameter estimation procedures as unknown parameters. However, this can be intractable as more nuisance parameters are involved in the estimation routine. An appealing approach is to fix initial conditions and estimate only the model parameters, e.g., [3, 21, 27]. In the target cell model, it is reasonable to assume that the number of infected cells at the beginning of infection is zero. However, the initial number of target cells and inoculum are experiment-dependent and can only be roughly approximated, e.g., initial inoculum had been chosen as half of lod [45].

**Fig 3:**
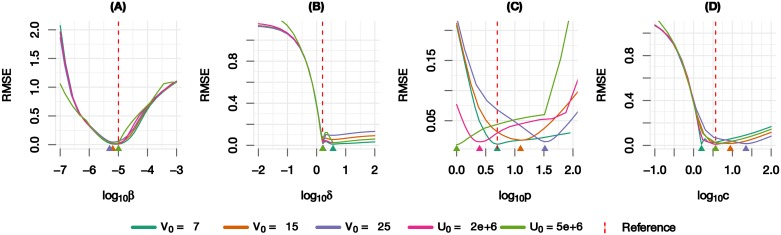
Parameters profiles in different initial conditions. The vertical dashed lines indicate the reference values, the triangles are the point estimated for the corresponding initial condition. The data include thirty replicates per time point with minimal measurement error, samples are collected in every three hours during 12 days of the experiment.

To isolate the effect of initial conditions on parameter estimates, an idealistic scenario was generated (Fig 2-Case 1, Table 1.a) and the model was fitted with the de algorithm as described in S1 Text. When the correct initial conditions of the model were used, the de algorithm returned the exact values of the parameters (S1 Fig). Afterwards, we varied the initial value of each component in the model and re-estimated the parameters and the corresponding profile likelihood. Because the data and the estimation algorithm did not changed, any error emerging in the parameter estimates can be attributed solely to the changes in the initial conditions. The tested values of the initial conditions were chosen to reflect biological range and its uncertainty in practice, i.e., the initial viral load were under the lod while the number of the initial target cells were about 10^7^ (Fig 2). Simulation results show that any deviations, even those that seem negligible, result in erroneous parameter estimates (Fig 3). Arbitrary choices of the initial viral load lead to very different estimates of essential viral infection kinetics parameters, e.g., both the replication rate *p* and the viral clearance *c* were estimated six times higher than the true value when fixing the initial viral load at half lod. Furthermore, subjective choices of model initial conditions result in flatness in the profile likelihood and shift the minimum in the parameters space. Note that the cost function values, i.e., rmse in this case, of different parameter sets are still almost identical.

Smoothing methods provide efficient ways to illustrate data dynamics. More importantly, they are free from the straitjacket of distributional assumptions and model structure [46]. As such, smoothing techniques could be potentially useful in providing estimates for missing data points and assisting the model parameter estimation. In this context, we tested the General Additive Model (GAM) [38, 47] to extrapolate the initial viral load value in numerous scenarios with a thousand simulations each. Each scenario was a combination of a sampling time scheme and a number of replicates per time point (Table 1.f). Simulation results show that GAM returns estimates for *V*_0_ ranging from 4 to 25 (S3 Fig). Although the range of estimated values is close to the reference value (*V*_0_ = 10), this approach may not sufficiently contribute to solving the problem of initial conditions and the subsequent error in parameter procedures.

### Measurement error impairs the parameter estimates

The previous scenario assumed virtually no measurement error whilst experimental data in practice can often be seen to vary in one to three orders of magnitude, e.g., see [11, 21, 48]. This is a fundamental barrier in learning from experimental data which will likely not be overcome in the near future. In this context, varying the imposed measurement error of synthetic data and profiling the parameters can provide useful hints on the possible influence of measurement error on parameter estimates. Using the correct initial conditions, an idealistic data set with a large number of replicates and dense sampling time was developed to isolate the effect of measurement error on the accuracy of parameter estimates (Table 1.b). Parameter estimation was done with the de algorithm as it had recovered the exact parameters when there were minimal measurement errors (S1 Fig). As expected, the results show that the estimates drift away from the reference value as the measurement errors increase (S2 Fig). More importantly, increasing the measurement error brings in more flatness to parameter profiles, implying difficulties in estimating parameter values with accuracy.

### Data asynchrony generates overlooked measurement error

Experimental data are usually taken for granted as it is observed timely. However, due to different reasons, e.g., host factors, experimental settings, measurement techniques, the observed data are likely to become *asynchronous*. In such situations, measurements recorded at one time point are at best an aggregate measure of the neighbour period.

**Fig 4:**
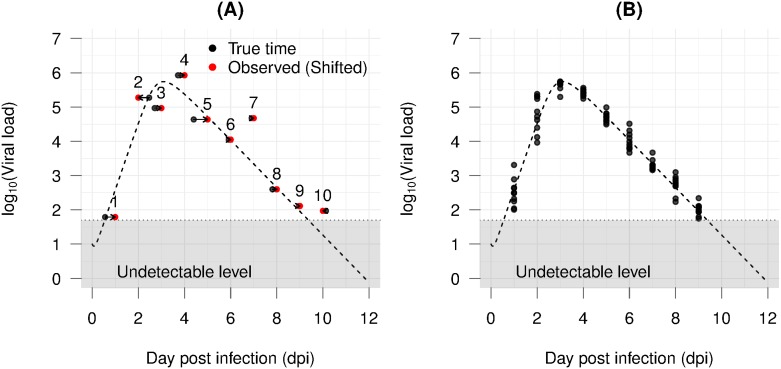
Examples of asynchrony problem. The dotted curve is the true kinetics, dots are data points. (A) Ten subjects with measurement error and asynchrony; red points are the observed data; black points are the actual time the measurement should have represented; arrows indicate directions of time shift; (B) Generated asynchrony data in the absence of measurement error.

The problem stated above can be coined the same as Berkson error type in statistics, where reported measurements are deviations of unknown true values [49]. Let *t* denote the real time point when an observed value actually happens; the time point will be instead recorded as $t^{*}=t+v,v∼N\left(0,\sigma_{t}^{2}\right)$, where *v* is an unknown time shift. This shifting could be attributed to the differences in subject-specific responding time to a stimulus and its strength, e.g., infection and the virulence of a virus strain. Furthermore, it is practically not feasible to infect all experimental subjects at the same time or to harvest all required replicates at once. For instance, subject one (Fig 4-A) for unknown reasons has ten hours delay in response to infection, but it is harvested at day first in the *experimental time scale*. This shifting underestimates the viral load on the first day by one order of magnitude.

By and large, the asynchrony leads to data with significant variations even in the absence of measurement error (Fig 4-B). To our knowledge, the scope of this problem has not been addressed or discussed in mathematical modelling works.

Simulations and extrapolation approaches had been shown to be an intuitive way to cope with Berkson error type in bioassay non-linear models [50], and seems to be a promising approach in dealing with data asynchrony in this context. We adapted the approach in an attempt to account for the data asynchrony problem as presented in S4 Text. However, applying simulation steps resulted in a non-linear pattern of parameter estimates (S4 Text). Consequently, it is not possible to make a proper extrapolation to estimates where the parameters are theoretically free from data asynchrony. This result opens a new challenge in mathematical modelling especially for biological processes with fast dynamics.

### Sparse and scarce data aggravate the parameter estimates

Data sampling in experimental settings is laborious and costly, consequently, experimental data are usually sparse and scarce. In iav, for example, the viral load had been measured in time intervals as short as eight hours [48] up to four days apart [51]. In each time point, there could be only a single replicate [52, 53] or up to sixteen replicates in rare cases [51]. In this context, investigating the impact of different data sampling schemes on parameter estimates provides further angles on how prone to error the parameter values can be in practice.

**Fig 5:**
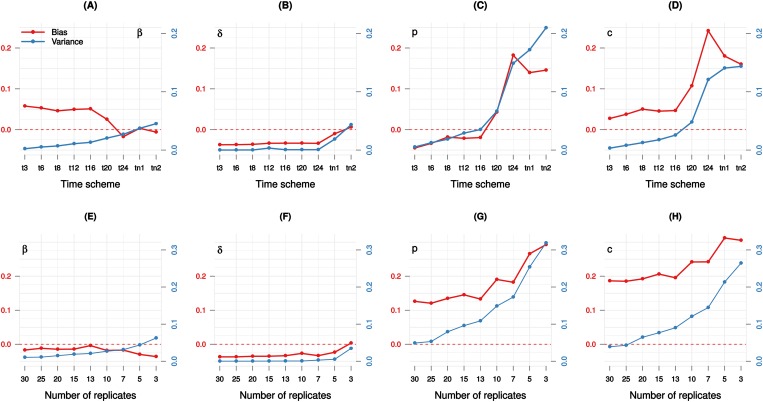
Sampling time scheme and parameter bias-variance. **Top**: t3 to t24 indicate regular sampling time in every 3 to 24 hours respectively; tn1 indicates sampling time similar to that of [11]; tn2 is t24 without measurement at the peak day (3rd) and the endpoint (8th). Ten replicates were generated per time point. Each scenario (point) is the result of 1000 simulations. **Bottom**: The every day sampling scheme (t24) is used.

To explore the impact of the sampling time scheme in more practical settings, scenarios in which the data are collected at various time intervals were generated, assuming ten replicates per time point with realistic measurement errors (Table 1.c). For each scenario, the estimation using the de algorithm was repeated on a thousand generated datasets to calculate bias and variance. Unsurprisingly, the parameter estimates variance increases as the sampling time gets sparser (Fig 5A–5D). Bias-variance of both parameters *p* and *c* escalates from 16h time scheme to 20h time scheme. It can be observed in Fig 5 that more precision is obtained by increasing the sampling time frequency, but little is gained in parameter accuracy. The practical experiment sampling time tn1 (6 time points and 5 replications) and tn2 (sampling every day but missing the peak day and last time point) manifest the highest variance (Fig 5).

Analysis of the number of replicates per time point was also examined in a similar fashion. The every day sampling scheme (t24) was chosen to see if the estimates could be improved by changing the number of replicates per time point (Table 1.d). As expected, the variance of most parameters increases when the number of measurements per time point is reduced, as well as the bias (Fig 5E–5H). There is not much gain in parameter accuracy as the number of replicates per time point roughly doubles from 13 to 30, and even less so the parameters *β* and *δ*.

Experimental design exhibits a strong influence on the magnitude of the bias, i.e., varying either the sampling time or the number of replicates per time point yield steep changes in bias magnitude. It seems that experimental design rewards more accuracy in parameter estimates by sampling more frequently rather than sampling more extensively (Fig 5).

It is worth noting that the examples thus far have shown that some model parameters were always estimated with larger error than the others given the same context (Figs 3, 5 and S2 Fig). This underlines the discrepancy of parameter accuracy level in a model, i.e., differences of parameter roles in a model lead to different levels of parameter accuracy.

### Parameter estimation algorithms agree on erroneous estimates

Thus far the de algorithm has shown that it can recover the exact parameters in an idealistic case. However, using a wrong assumption for the model or poor experimental data can both drive the algorithm to the wrong estimates. With the progress in parameter estimation techniques these days, it would be intriguing to know whether there is an algorithm that can recover more accurate estimates given a realistic experimental data set. It has been shown that there is not a definitive algorithm that is recommended for all situations [54]. To this end, we challenged the state-of-the-art algorithms to estimate the four model parameters in a practical context (Fig 2-Case 1). We assume that the initial conditions are known to make the parameters structurally identifiable [9] (Table 1.e). The tested algorithms include a box-constraint local estimation algorithm, i.e., L-BFGS-B) [44], a global stochastic algorithm i.e., Differential Evolution (DE) [43], and a Bayesian sampling approach, i.e., Adaptive Metropolis-Hasting (MH) [55]. Maximum likelihood estimation (MLE) and least square (LS) estimation were considered where relevant to reflect their usage in practices. To avoid imposing subjective bias in favour of one algorithm, the algorithms were supplied with their recommended settings (S1 and S2 Texts).

**Fig 6:**
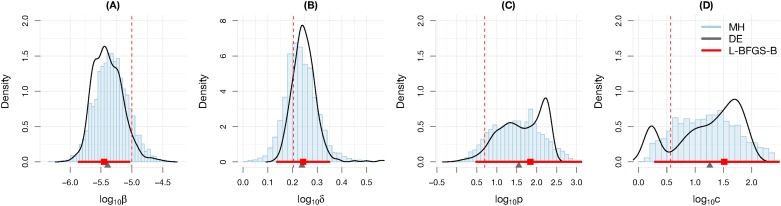
Parameter estimates using different algorithms. The data are collected similar to [11] at day 1, 2, 3, 5, 7, 9, five replicates per time point. **MH**: Metropolis–Hasting estimates are presented as blue histograms of posterior samples; **L-BFGS-B**: local optimization and confidence intervals estimated with approximate Fisher information are presented with red square points and bars; **DE**: point estimates of the global optimization algorithm Differential Evolution and weighted bootstrapping samples are presented with black triangles and curves. The vertical red dashed line is the reference value.

Simulation results show that the algorithms return relatively similar, whilst inaccurate point estimates of the parameters, e.g., the two parameters *p* and *c* are distanced by one order of magnitude from the correct ones (Fig 6). Interval estimates of the three algorithms cover similar ranges that spread up to two orders of magnitude. Good interval estimates can be obtained only for the parameter *δ*. Note that the bootstrapping samples exhibit multi-modal curves, implying there there is at least one outlier in the used data [56]. Given the same advantages, the intensive estimation processes do not reward better estimates, i.e., the L-BFGS-B converged in a few minutes, the mh took twenty minutes to converge to a stable distribution (S2 Text) and the bootstrapping with the de algorithm took a day on a high-performance computer with parallel mode enabled (S1 Text).

The estimations above were conducted in very favourable conditions that are unlikely to occur in practice, i.e., correct model of the observed data, known initial condition values, real parameter values are in the ranges of searching parameter spaces. Even with those advantages, the parameter values are risky to be interpreted, e.g., the viral replication rate (*p*) can be recognized as much as one hundred copies per day with strong supports from the interval estimates of different algorithms. Certainly, expecting more accurate estimates in practice are rather overoptimistic, especially when most often none of the above advantages exist in practice. Noting that the same settings for the de algorithm has been shown to be able to recover the exact parameters in an idealistic data set (S1 Fig) and the mh algorithm had converged to a stable distribution (S2 Text). In addition, a test for different combinations of de’s algorithm parameters was conducted showing that tuning these DE algorithm’s parameters did not improve the rmse (S4 Fig). Thus, it can be inferred that the main force that has driven algorithms to the wrong estimates was the poor experimental setting.

This example also suggests an influential role of outliers in estimating mathematical model parameters. Sources of outliers data could be merely originated from measurement errors or, in fact, come from a different generating mechanism. Robust correction is a popular approach to scale down the influences of those data points by assigning them lesser weights in the optimization process and is believed to reduce their impact on parameter estimates [57]. To test this approach in a model based on ode, thousands of synthetic data sets with outliers were generated. Then the estimation procedures with and without correction for outliers were applied to each of the data sets (S3 Text, Table 1.g). The results show that correction for outliers in this model did not improve the accuracy of the parameter estimates (S3 Text) while the computational cost increased as several rounds of optimization may be needed to achieve stable weights in robust estimation.

## Discussion

Mathematical modelling research comprises several disciplines with mutual concepts, approaches, and techniques. Significant concerns about the validity of parameter values had emerged when a majority of modelling works failed to mention the risks of errors in the parameter values while trying to extract biological meaning from it. Throughout this paper, by presenting numerical examples and addressing their implications, we explored how error-prone the parameter estimates are in everyday practices.

It is a catch-22 situation when the way we express a parameter in the model governs its own accuracy. The results show that given the same context, there are model parameters which can be estimated with less error while the others cannot. This reflects the reality that the corresponding component of a parameter in the model is less important in the dynamics. To provide assessments in this aspect, one can head for a global sensitivity analysis approach [58, 59]. However, in biological systems, one can expect joint effects of parameters on the system’s output [14]. This leads to situations in which an inaccurate set of parameters generates the same dynamics as the correct one. In this case, the estimated parameter values need to be taken with caution as they are a possible solution in a pool of model solutions. Altogether, in the worst case, the model structure alone already hinders the parameter accuracy, making the interpretation of the parameter values questionable. Reporting assessments of model parameters sensitivity, parameters correlation and identifiability, e.g., [14, 35], can avoid the model results to be over-interpreted.

Leaving out the validity of a model to represent a certain phenomenon, parameter estimation of a correct model with known parameters and fabricated data exhibited several shortcomings. Each algorithm has *per se* a number of parameters as well as configurations that are required to be specified by the user. The way we choose these settings may directly affect the estimation results. For instance, algorithms might be trapped in a local optimum and this can be tested by tuning the algorithm. Although assessing this aspect is beyond the scope of the work proposed here, simulation results support that the recommended setting is among the best ones for the DE algorithm (S4 Fig). That is why all the algorithms configurations were set based on the literature recommendations as described in S1, S2 and S3 Texts. In addition, testing different algorithms for a study case can help to find out if one algorithm was trapped in a local minimum. However, even in ideal conditions, our results show that different algorithms returned erroneous estimates. Note that the model parameters in the tested situation were set to be theoretically identifiable. This result poses a big question mark on the validity of mathematical model parameters in practice, where the models are more complex but the known model conditions and experimental data are limited.

In accordance with [54], we observed that there is not a conclusive method for parameter estimation. Advanced Bayesian sampling techniques have been developed recently [60], however, these are neither practical to implement in complex mathematical models nor beneficial for parameter accuracy but can serve to explore the posterior distributions. In this family, the Adaptive mh algorithm [55] seems more practical and less dependent on model complexity but it lacks dedicated resources for mathematical modelling (S2 Text). The bootstrap estimates exhibited its shortcoming when the data contained outliers and required more work in modelling context. An example of tackling the outliers based on robust correction approaches also did not show any improvement in parameter accuracy compared to the least square estimation (S3 Text). By and large, one should not expect the power of innovative fitting algorithms to handle sparse and scarce data and to return trustworthy estimates.

Parameters have been deemed reliable when parameter profiles exhibit convex shape [13, 35]. However, our results show that reaching the minimum of the parameter profile curve does not imply that the estimate is accurate (Fig 3). The results show that the revealed parameters are noticeably different from the simulated values regardless that the optimization algorithm has successfully found the minima on the parameter profile curves. Thus, one should take this convergence criterion with caution when attempting to extract biological information, even if one’s favourite algorithm works.

In experimental settings, several sources of errors exist that are difficult to overcome. In the target cell model, for example, it is not an easy task for experimentalists to define the exact initial conditions even with the advance of laboratory devices. In contrast, mechanistic models work with a relatively high precision level. This is a straitjacket for all modellers. Working with assumed ode model initial conditions might be the first choice and could be a valid one if the aim is not biological parameter quantification. This approach reduces the number of parameters to estimate in the shortage of data and may hold up in model prediction contexts. However, our results show that the estimates went wrong with seemingly reasonable choices of initial conditions in an idealistic experimental data set (Fig 3). Reporting biological parameters from a mathematical model should incorporate the uncertainty assessment regarding the initial conditions used, for example, a sensitivity analysis with respect to initial conditions.

Smoothing techniques are attractive in mathematical modelling as they can provide a generic portrayal of the dynamics without knowing the underlying mechanism. For instance, the fractional polynomial model was used to estimate the initial viral load [35]. The generalized smoothing approach and local polynomial regression have also been proposed for estimating parameters [61, 62]. However, there is a caveat observed in this paper when modelling with extrapolated data that is most likely to exist in others. Our example of using smoothing techniques to estimate the initial viral load showed that the sparser data tend to have the lesser bias in extrapolating the initial viral load (S3 Fig). All sampling time schemes that collect data regularly in less than 24 hours exhibit a consistent positive error and *vice versa*. This can be attributed to the fact that all the measurements below lod are removed when generating the data to reflect the undetectable level in practice. The loss of information from those data points under the lod in combination with measurement errors makes it more likely that the observed data at the early, as well as the later time points, of the dynamics could be observed higher than it is. Accordingly, making extrapolation to the time point zero, or to the latest time points can result in an overestimation. This result suggests that fitting the model to a smoothed line could potentially skew the parameter estimates.

We introduced an abstraction of the *data asynchrony* and studied its impact on experimental data which, ultimately, affects the parameter estimates. To the best of our knowledge, this issue has been widely ignored in mathematical modelling. Segregating the measurement error and the errors induced by asynchrony seems impossible. A logical approach is to simulate the amount of time shift and examine its effect on model parameters. In S4 Text we presented an attempt to deal with data asynchrony by simulation and extrapolation to facilitate the conversations in the field. However, further efforts are needed to resolve or, at least, to account for the impact of the asynchrony in mathematical modelling results.

Simulation results show that insufficient data lower the quality of the parameter estimates. In contrast, it is not necessary to collect data extensively, but rather rationally to reduce the error. An experimental design approach suggesting that accurate parameter estimation is achievable with complementary experiments specifically designed for the task [63, 64]. A somewhat simpler version of this idea is that reducing error in parameter estimates can be achieved by fully measuring the model components [23]. Altogether, this supports the fundamental idea as shown in our examples: the primary source to improve the quality of parameter estimates is additional data, in particular, collected in a way designated for a modelling purpose.

The scientific community can actively benefit from an advanced understanding of the sources of error. The purpose of spotting these sources is to avoid or at least to recognize them. Efforts in dealing with errors in parameter estimation shall be documented in publications with mathematical models to strengthen and support further development in the field. There are rooms for further refinement of estimation methodologies to minimize the risk of reporting erroneous estimates and different types of measurement errors in experimental studies. Above all, however, there is a need to unify the efforts in modelling practices, such as develop good practice guidelines for reporting mathematical model assessment results, communicate the difficulties, and construct a common software library. Ultimately, a reassessment of the current approach in modelling experimental data might be needed to shape the future research on a more solid path, maintaining trust in the scientific community of the diligent works in modelling biological systems.

## Supporting Information

S1 Text Limited-memory BFGS (L-BFGS-B) and Differential Evolution (DE) algorithm settings. (PDF)

S2 Text Implementation of Adaptive Metropolis-Hasting in ODE models. (PDF)

S3 Text Implementation of robust estimation in ODE models. (PDF)

S4 Text Implementation of simulation and extrapolation approach. (PDF)

S5 Text Description of search results from the PubMed Central database. (PDF)

S1 Fig Parameters profiles in an idealistic scenario. The vertical dashed lines indicate the reference values, the solid circles are the estimated values from Differential Evolution algorithm. The data include thirty replicates per time point with minimal measurement error, i.e., ∼*N*(0, 0.01) on log base ten, samples are collected in every three hours during 12 days of the experiment (Fig 2-Case 2).
(EPS)

S2 Fig Parameter likelihood profiles in different imposed measurement error. The solid triangles indicate the reference values, the circles are the estimated values corresponding to each condition. Parameters are presented in log base 10 scale. The imposed errors are approximately one to two orders of magnitude of viral load measurements. The profiles lines are standardized by their imposed measurement error for visual purposes.
(EPS)

S3 Fig Bias-variance of *V*_0_ estimates in different scenarios. Each scenario (point) is the result of 1000 simulations. The legends t3 to t24 indicate a regular sampling time every 3 to 24 hours; tn1 indicates sampling time similar to that of [11]; tn2 is t24 without measurement at the peak day (3rd) and the endpoint (8th).
(EPS)

S4 Fig RMS values versus *F* and *CR* parameters combination of de algorithm. Colours indicate the corresponding values of *CR*. The plus sign indicates the value that is used in this paper.
(EPS)
