# Supplementary material for: Analysis of Practical Identifiability of a Viral Infection Model
Source: PLoS One. 2016 Dec 30;11(12):e0167568. doi: 10.1371/journal.pone.0167568 (PMC5201286; doi:10.1371/journal.pone.0167568)
Supplement: S1 Text — (PDF) [file pone.0167568.s001.pdf]

Analysis of practical identifiability of a viral infection model

Van Kinh Nguyen[1,2,3], Frank Klawonn[4,5], Rafael Mikolajczyk[6,7,8,9], Esteban A. Hernandez-Vargas[1,*]

1 Systems Medicine of Infectious Diseases, Department of Systems Immunology and Braunschweig Integrated Centre of Systems Biology, Helmholtz Centre for Infection Research, Braunschweig, Germany, **2** Epidemiology Department, Ho Chi Minh University of Medicine and Pharmacy, Ho Chi Minh, Vietnam. **3** PhD Programme “Epidemiology”, Braunschweig-Hannover, Germany **4** Biostatistics, Helmholtz Centre for Infection Research, Braunschweig, Germany, **5** Department of Computer Science, Ostfalia University, Wolfenbüttel, Germany, **6** Epidemiological and Statistical Methods, Helmholtz Centre for Infection Research, Braunschweig, Germany. **7** German Centre for Infection Research, site Hannover-Braunschweig, Germany **8** Hannover Medical School, Hannover, Germany **9** [Institute of] Medical Epidemiology, Biometry and Informatics, Martin-Luther University Halle-Wittenberg, Germany

* esteban.vargas@helmholtz-hzi.de

S1 Text

Limited-memory BFGS (L-BFGS-B) algorithm settings. Maximum likelihood estimation was done with the L-BFGS-B algorithm implemented in R [2]. The parameter boundaries are shown in Table 1 (main text). The relative tolerance was set at 10^{-8}. The parameter estimation was conducted in log base ten for both the states and parameters.

Differential Evolution (DE) algorithm settings. The parameter estimation was conducted in log base ten for both the states and parameters. The parameter boundaries are shown in Table 1 (main text). Root mean squared error (RMSE) was used as the cost function. The DE algorithm was run with recommended configurations from [1]. In particular, set the number of parents NP to 10 times the number of parameters (40), select differential weighting factor $F = 0.8$ and crossover constant $CR = 0.9$. A consecutive thirty iterations without an improvement in the root mean squared error (RMSE) (10^{-8}) will stop the optimizer. Note that the maximum number of iterations needs to be set to an arbitrarily large number (10^4) to prevent DE algorithm stops early in problematic data. The algorithm was run in parallel mode. The weighted bootstrapping was done the same as described in [3], i.e., assigning to the cost function a vector of random weights with length equal the sample size from the exponential distribution with mean one and variance one.

References

1. Storn R, Price K. Differential Evolution – A Simple and Efficient Heuristic for global Optimization over Continuous Spaces. Journal of Global Optimization. 1997;11(4):341–359.
2. R Core Team. R: A Language and Environment for Statistical Computing. Vienna, Austria; 2014.
3. Ma S, Kosorok MR. Robust semiparametric M-estimation and the weighted bootstrap. Journal of Multivariate Analysis. 2005;96(1):190 – 217.
