# Supplementary material for: Analysis of Practical Identifiability of a Viral Infection Model
Source: PLoS One. 2016 Dec 30;11(12):e0167568. doi: 10.1371/journal.pone.0167568 (PMC5201286; doi:10.1371/journal.pone.0167568)
Supplement: S2 Text — (PDF) [file pone.0167568.s002.pdf]

Analysis of practical identifiability of a viral infection model

Van Kinh Nguyen[1,2,3], Frank Klawonn[4,5], Rafael Mikolajczyk[6,7,8,9], Esteban A. Hernandez-Vargas[1,*]

1 Systems Medicine of Infectious Diseases, Department of Systems Immunology and Braunschweig Integrated Centre of Systems Biology, Helmholtz Centre for Infection Research, Braunschweig, Germany, **2** Epidemiology Department, Ho Chi Minh University of Medicine and Pharmacy, Ho Chi Minh, Vietnam. **3** PhD Programme "Epidemiology", Braunschweig-Hannover, Germany **4** Biostatistics, Helmholtz Centre for Infection Research, Braunschweig, Germany, **5** Department of Computer Science, Ostfalia University, Wolfenbüttel, Germany, **6** Epidemiological and Statistical Methods, Helmholtz Centre for Infection Research, Braunschweig, Germany. **7** German Centre for Infection Research, site Hannover-Braunschweig, Germany **8** Hannover Medical School, Hannover, Germany **9** [Institute of] Medical Epidemiology, Biometry and Informatics, Martin-Luther University Halle-Wittenberg, Germany

* esteban.vargas@helmholtz-hzi.de

S2 Text

Implementation of Adaptive Metropolis-Hasting in ODE models. A realistic dataset with five replicates per time point and collection time scheme as in [1] was generated. The model was parameterized in log 10 space. A multivariate normal distribution $N(\theta, c^2\Sigma)$ and a uniform prior $U(0,1)$ are used as proposals for the model parameters and measurement error, respectively. The target acceptance rate for measurement error and the four model parameters is 0.45 and 0.35 [4], respectively. The initial scale of the model parameters covariance matrix is $c^2 = 2.38^2/d$, where $d = 4$ is the number of parameters [4]. The following tuning steps [5] are done during the tuning phase to reach within a small tolerance of the target acceptance rate (± 0.075): (1) The scale parameter c is adjusted as a function of the observed and the target acceptance rate as in [2, 3], (2) the covariance matrix is tuned by taking the weighted average of the covariance matrix observed between one loop to the previous. The weight of 0.75 is chosen to put more weight on the observed one, (3) burn-in period of size 5000 and 1000 posterior samples are used resulting in 5000 samples (thinning rate is 10). Three MCMC chains starting from different proposal values are run. Four tuning loops with five hundred iterations each are needed to reach the target acceptance rate. The code skeleton can be found on-line at this link.

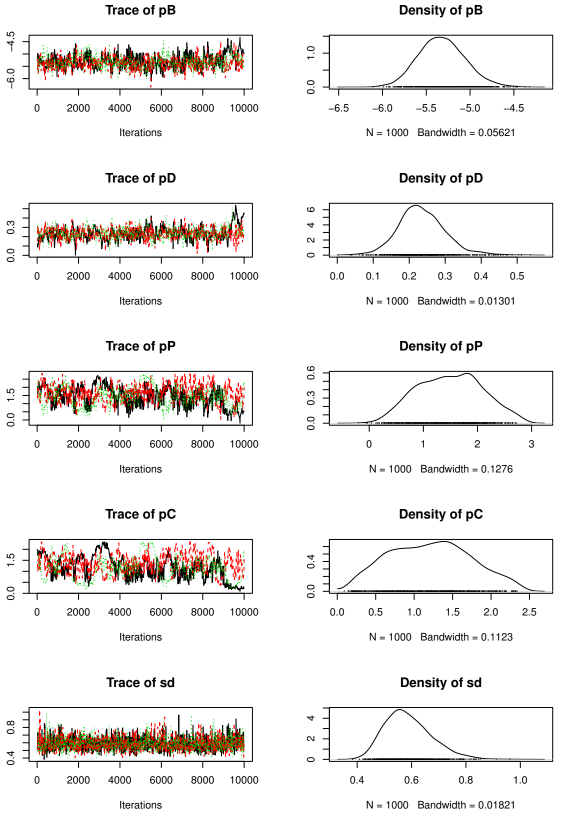

Figure T2. Trace-plots and posterior density of the four parameters in the target cell model. Applying Metropolis–Hasting (MH) in a realistic dataset with five replicates per time point and collection time scheme as in [1]. The pB, pD, pP, pC, and sd are β, δ, p, c and the standard deviation of the imposed measurement error. The adaptive Metropolis-Hasting algorithm is used.

References

1. Toapanta FR, Ross TM. Impaired immune responses in the lungs of aged mice following influenza infection. Respir Res. 2009;10:112.
2. Roberts GO, Gelman A, Gilks WR. Weak convergence and optimal scaling of random walk Metropolis algorithms. Ann Appl Probab. 1997 02;7(1):110–120.
3. Roberts GO, Rosenthal JS. Optimal scaling for various Metropolis-Hastings algorithms. Statist Sci. 2001 11;16(4):351–367.
4. Gelman A, Carlin JB, Stern HS, Dunson DB, Vehtari A, Rubin DB. Bayesian Data Analysis, Third Edition. Chapman & Hall/CRC Texts in Statistical Science. Taylor & Francis; 2013.
5. Haario H, Laine M, Mira A, Saksman E. DRAM: Efficient adaptive MCMC. Statistics and Computing. 2006;16(4):339–354.
