# Supplementary material for: Analysis of Practical Identifiability of a Viral Infection Model
Source: PLoS One. 2016 Dec 30;11(12):e0167568. doi: 10.1371/journal.pone.0167568 (PMC5201286; doi:10.1371/journal.pone.0167568)
Supplement: S3 Text — (PDF) [file pone.0167568.s003.pdf]

Analysis of practical identifiability of a viral infection model

Van Kinh Nguyen[1,2,3], Frank Klawonn[4,5], Rafael Mikolajczyk[6,7,8,9], Esteban A. Hernandez-Vargas[1,*]

1 Systems Medicine of Infectious Diseases, Department of Systems Immunology and Braunschweig Integrated Centre of Systems Biology, Helmholtz Centre for Infection Research, Braunschweig, Germany, **2** Epidemiology Department, Ho Chi Minh University of Medicine and Pharmacy, Ho Chi Minh, Vietnam. **3** PhD Programme "Epidemiology", Braunschweig-Hannover, Germany **4** Biostatistics, Helmholtz Centre for Infection Research, Braunschweig, Germany, **5** Department of Computer Science, Ostfalia University, Wolfenbüttel, Germany, **6** Epidemiological and Statistical Methods, Helmholtz Centre for Infection Research, Braunschweig, Germany. **7** German Centre for Infection Research, site Hannover-Braunschweig, Germany **8** Hannover Medical School, Hannover, Germany **9** [Institute of] Medical Epidemiology, Biometry and Informatics, Martin-Luther University Halle-Wittenberg, Germany

* esteban.vargas@helmholtz-hzi.de

S3 Text

Implementation of robust estimation in ODE models. A viral load data set collected daily with ten replicates per day is generated. Outliers data points are added by sampling from a skew normal distribution which resulted in outliers different in one order of magnitude; both left- and right-skew outliers are considered, i.e., skew parameter $\alpha = \pm 10$. The robust estimation steps are then conducted as in [1]: (1) Obtain the parameter estimates using the Least squares (LS) method, scale the residuals with mean absolute deviation from the median (MAD); (2) Compute Huber weights [2] based on the scaled residuals; (3) Optimize model with weighted least squares (WLS) using Huber weights, compute new scaled residuals; (4) Iterate steps 2-3 until the maximum changes in Huber weights from one iteration to the previous is not greater than a small threshold, e.g., 0.05; (5) Repeat steps 2-4 but instead using Tukey's bisquare weight function and a smaller convergence threshold, e.g., 0.01. The code skeleton can be found on-line at this link. There are high demands of computational cost but there is virtually no gain in parameter accuracy of the robust estimates compared to the normal LS estimates (Fig. T3).

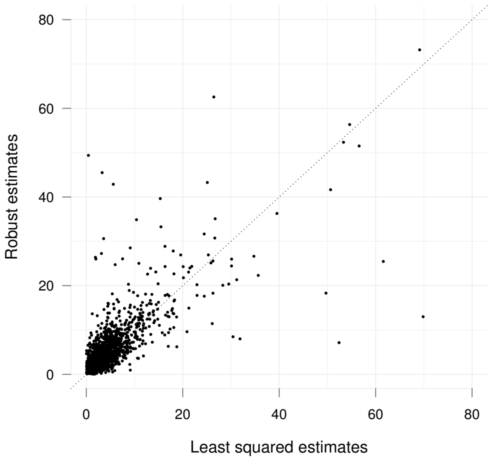

Figure T3. Robust vs. least squares estimates. Robust and LS are tested on 2000 datasets with outliers. Mahalanobis distance from each set of parameters to the reference one is presented.

References

1. Li G. In: Robust Regression. John Wiley & Sons, Inc.; 2006. p. 281–343.
2. Huber PJ, Ronchetti EM. Robust Statistics. Wiley Series in Probability and Statistics. Wiley; 2009.
