# Supplementary material for: Analysis of Practical Identifiability of a Viral Infection Model
Source: PLoS One. 2016 Dec 30;11(12):e0167568. doi: 10.1371/journal.pone.0167568 (PMC5201286; doi:10.1371/journal.pone.0167568)
Supplement: S4 Text — (PDF) [file pone.0167568.s004.pdf]

Analysis of practical identifiability of a viral infection model

Van Kinh Nguyen[1,2,3], Frank Klawonn[4,5], Rafael Mikolajczyk[6,7,8,9], Esteban A. Hernandez-Vargas[1,*]

1 Systems Medicine of Infectious Diseases, Department of Systems Immunology and Braunschweig Integrated Centre of Systems Biology, Helmholtz Centre for Infection Research, Braunschweig, Germany, **2** Epidemiology Department, Ho Chi Minh University of Medicine and Pharmacy, Ho Chi Minh, Vietnam. **3** PhD Programme "Epidemiology", Braunschweig-Hannover, Germany **4** Biostatistics, Helmholtz Centre for Infection Research, Braunschweig, Germany, **5** Department of Computer Science, Ostfalia University, Wolfenbüttel, Germany, **6** Epidemiological and Statistical Methods, Helmholtz Centre for Infection Research, Braunschweig, Germany. **7** German Centre for Infection Research, site Hannover-Braunschweig, Germany **8** Hannover Medical School, Hannover, Germany **9** [Institute of] Medical Epidemiology, Biometry and Informatics, Martin-Luther University Halle-Wittenberg, Germany

* esteban.vargas@helmholtz-hzi.de

S4 Text

Implementation of simulation and extrapolation approach. Let t denote the true time point when an observed value actually happens; the corresponding time point in experimental scale is $t^* = t + v, v \sim N(0, \Sigma_v)$, where v is an unknown time shift. The following steps are done (adapted from [1]): (1) Define Σ_v such that t varies in a biological range according to the model; (2) Generate a sequence of λ_i in $\{0, ..., K\}$; (3) For each λ_i, add time shifts to the original data with the noise equal to $\lambda_i \Sigma_v$ and fit the model. The new time shift for each data point thus is equal to $(1+\lambda_i)\Sigma_v$; (4) Repeat step 3 B times (B sufficiently large) and obtain the average parameter estimates for each λ_i; (5) Use statistical models to make extrapolation of parameter values at $\lambda = -1$.

In the IAV kinetics, the shift v could be assumed varying from a few minutes to a day which can be illustrated by a truncated normal distribution. The λ sequence length of ten and $K = 1$ are chosen such that the simulated data have realistic time scale and maximum time shift of one day, $B = 1000$ simulations is used. The results showed that applying simulation step in the model resulted in a non-linear pattern of parameter estimates over λ (Fig. T4). Thus it is not possible to make extrapolation properly to where theoretically no error at $\lambda = -1$.

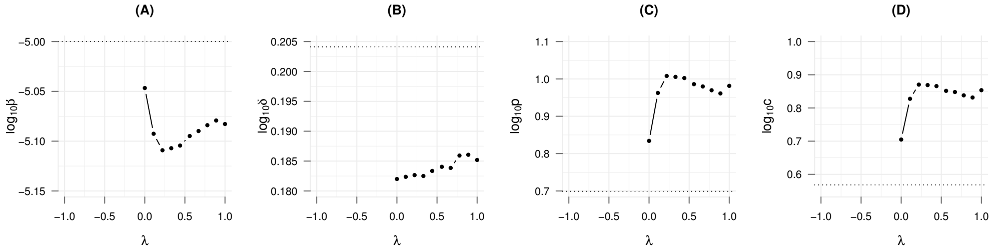

Figure T4. Simulation and extrapolation approach in the target-cell limited model. The horizontal dashed line corresponds to the reference values, each dot is the mean of 1000 simulations of time shifts.

References

1. Althubaiti A, Donev A. Non-Gaussian Berkson errors in bioassay. Statistical Methods in Medical Research. 2012;.
