# Supplementary material for: Analysis of Practical Identifiability of a Viral Infection Model
Source: PLoS One. 2016 Dec 30;11(12):e0167568. doi: 10.1371/journal.pone.0167568 (PMC5201286; doi:10.1371/journal.pone.0167568)
Supplement: S5 Text — (PDF) [file pone.0167568.s005.pdf]

Analysis of practical identifiability of a viral infection model

Van Kinh Nguyen[1,2,3], Frank Klawonn[4,5], Rafael Mikolajczyk[6,7,8,9], Esteban A. Hernandez-Vargas[1,*]

1 Systems Medicine of Infectious Diseases, Department of Systems Immunology and Braunschweig Integrated Centre of Systems Biology, Helmholtz Centre for Infection Research, Braunschweig, Germany, **2** Epidemiology Department, Ho Chi Minh University of Medicine and Pharmacy, Ho Chi Minh, Vietnam. **3** PhD Programme "Epidemiology", Braunschweig-Hannover, Germany **4** Biostatistics, Helmholtz Centre for Infection Research, Braunschweig, Germany, **5** Department of Computer Science, Ostfalia University, Wolfenbüttel, Germany, **6** Epidemiological and Statistical Methods, Helmholtz Centre for Infection Research, Braunschweig, Germany. **7** German Centre for Infection Research, site Hannover-Braunschweig, Germany **8** Hannover Medical School, Hannover, Germany **9** [Institute of] Medical Epidemiology, Biometry and Informatics, Martin-Luther University Halle-Wittenberg, Germany

* esteban.vargas@helmholtz-hzi.de

S5 Text

Description of search results from the PubMed Central database. Using the syntax provided by PubMed Central [1], we conducted a search of scientific works on Pubmed Central database from 1990 to March 2016. The number of papers that used the keywords `ordinary differential equation(s)` *explicitly* in the paper formed the first category *ODEs*. By explicitly, we mean the search terms of interested *must* appeared in the title, *or* the abstracts *or* within the main text, *and* excluding those have it in references *or* if the paper is a review paper. To obtain the number of papers that conducted parameter estimation, the following keywords and their variation must be used explicitly, including `estimat*` *or* `quanti*`, *or* `fit*`. For the last category, the variations of the keyword `identifi*` must be used explicitly.

References

1. Pubmed Central. PMC Help - NCBI Bookshelf; 2016.
